# Serous Cyst in Superior Maxillary Sinus

**Published:** 1890-09

**Authors:** J. H. Frink


### SEROUS CYST IN SUPERIOR MAXILLARY SINUS.

By J. H. Frink, V.S.

In October I was asked to examine a highly-bred trotting foal, five months old, female, of unusual height, very large bones and joints, coarsely made, the trunk light in proportion to the extremities, in fair condition of nourishment. The pulse and temperature were normal. The animal was supposed to have some bronchial or throat trouble. When undisturbed, a slight wheezing could be easily detected in the respiration, but when disturbed or excited a loud whistling, sawing noise was produced quickly, which subsided when the animal remained quiet. The appetite was good, it had no soreness of the throat, nor cough; the eyes had a brilliant appearance, the head was carried in an easy, natural position; there was an intermittent, muco-purulent discharge from both nostrils, free on one day and hardly noticeable on another; the nostrils were dilated to their utmost capacity during respiration; absence of disease of the throat, bronchi or lungs, with dullness on percussion over the superior maxillary sinus, led to the diagnosis of obstruction there, which the owner would not accept.

The animal was treated with counter-irritants along the course of the trachea and sub-maxillary space and received febrifuges for some weeks, when I was again called in. At this time a decided enlargement was visible about three-quarters of an inch superior and anterior to the termination of the superior maxillary spine on the right side; it was irregular, ovoid, about one-and-a-half inches in length. I again gave as my diagnosis an obstruction of the superior maxillary sinus. I advised trephining, which the owner declined to accede to, preferring to wait awhile to see what course nature would pursue. Distention, or rather displacement, of the bones of the face from this time on was very rapid, with an enormous bulging over the sinus. The malar, lachrymal, and even the nasal bones shared in the general deformity. In January the superior maxillary spine was lost to view, and there was an excessive purulent discharge from the inner canthus of the eye; the latter was almost pushed from the orbit. The head produced the appearance of a monstrosity.

The appetite remained good, but the respirations were extremely loud and labored; a careful examination of the teeth was made, but no trouble could be found in them. Very little air passed through the nasal fossa on the diseased side. I was asked to operate; the animal was cast, partial anesthesia was attempted, but from the distressed and violent respiration it was thought best to desist. A circular incision was made at the most prominent part, at that part that the disease first manifested itself. When the flap had been lifted the bone presented a light brownish appearance. The bone was soft and friable, and the button split into several pieces. Upon opening the sinus a quantity of bright yellow fluid escaped, which afterward changed to a darker tint, resembling serum; after an injection of carbolic solution the animal was allowed to rise; when it got to its feet it threw its nose upward and an increased quantity of fluid escaped.

I deemed that I had made an error in not trephining at a lower point in the sinus, but the foal so constantly threw its head up that I saw that further operation was not necessary. In a few days there was a profuse purulent discharge from the nostrils with a barely noticeable fetor. This odor entirely subsided in a day or two; there was no modification of the noise made in breathing. At or about the twelfth day it was noticed that air was passing through the nasal fossa and the discharge had become less; the hair was denuded by the continued discharge. It became necessary to enlarge the opening made by the trephining; this was only accomplished with difficulty, as the animal was unbroken and hard to handle. At the expiration of six weeks there was a marked modification in the respiration and the discharge had nearly ceased; the opening was kept patulous with great difficulty and closed shortly, at the expiration of nine weeks. Noise on respiration was only perceptible when the animal was moved and it grew less every week. Four months afterward the bones of the face had gradually flattened and assumed their natural form. The animal was put to a fast trot ending in a gallop with no abnormal sound on either inspiration or expiration.

I consider the case an excellent illustration of a benign growth, terminating satisfactorily by proper treatment, which, if it had been left alone for a longer time, as desired by the owner, would have produced such caries in the bone and irritation in the surrounding tissues as to have produced an osteo-sarcoma, or such lesions as would have rendered it malignant. Again, at the outset it bore a certain resemblance to osteo-porosis, and it demanded prompt surgical handling to verify the diagnosis.

